# Consumption of vegetables and their relation with ultra-processed foods in Brazil

**DOI:** 10.11606/S1518-8787.2018052000111

**Published:** 2018-05-03

**Authors:** Daniela Silva Canella, Maria Laura da Costa Louzada, Rafael Moreira Claro, Janaina Calu Costa, Daniel Henrique Bandoni, Renata Bertazzi Levy, Ana Paula Bortoletto Martins

**Affiliations:** I Universidade do Estado do Rio de Janeiro. Instituto de Nutrição. Departamento de Nutrição Aplicada. Rio de Janeiro, RJ, Brasil; II Universidade de São Paulo. Núcleo de Pesquisas Epidemiológicas em Nutrição e Saúde. São Paulo, SP, Brasil; III Universidade Federal de São Paulo. Instituto de Saúde e Sociedade. Departamento de Políticas Públicas e Saúde Coletiva. Santos, SP, Brasil; IV Universidade Federal de Minas Gerais. Escola de Enfermagem. Departamento de Nutrição. Belo Horizonte, MG, Brasil; V Universidade Federal de Pelotas. Programa de Pós-Graduação em Epidemiologia. Pelotas, RS, Brasil; VI Universidade Federal de São Paulo. Instituto de Saúde e Sociedade. Departamento de Saúde, Clínica e Instituições. Santos, SP, Brasil; VII Universidade de São Paulo. Faculdade de Medicina. Departamento de Medicina Preventiva. São Paulo, SP, Brasil

**Keywords:** Industrialized Foods, Vegetables, economics, Food Consumption, Feeding Behavior, Diet, Food, and Nutrition, Diet Surveys, Alimentos Industrializados, Verduras, economia, Consumo de Alimentos, Comportamento Alimentar, Inquéritos sobre Dietas, Alimentos, Dieta e Nutrição

## Abstract

**OBJECTIVE:**

To characterize the household purchase and the individual consumption of vegetables in Brazil and to analyze their relation with the consumption of ultra-processed foods.

**METHODS:**

We have used data on the purchase of food for household consumption and individual consumption from the 2008–2009 Brazilian Household Budget Survey. The Brazilian Household Budget Survey studied the purchase of food of 55,970 households and the food consumption of 34,003 individuals aged 10 years and over. The foods of interest in this study were vegetables (excluding roots and tubers) and ultra-processed foods. We have described the amount of vegetables (grams) purchased and consumed by all Brazilians and according to the quintiles of caloric intake of ultra-processed food. To this end, we have calculated the crude and predicted values obtained by regression models adjusted for sociodemographic variables. We have analyzed the most commonly purchased types of vegetables (% in the total amount) and, in relation to individual food consumption, the variety of vegetables consumed (absolute number), the participation (%) of the types of culinary preparations based on vegetables, and the time of consumption.

**RESULTS:**

The adjusted mean household purchase of vegetables was 42.9 g/*per capita*/day. The adjusted mean individual consumption was 46.1 g. There was an inverse relation between household purchase and individual consumption of vegetables and ultra-processed foods. Ten types of vegetables account for more than 80% of the total amount usually purchased. The variety consumed was, on average, 1.08 type/*per capita*/day. Approximately 60% of the vegetables were eaten raw, and the amount consumed at lunch was twice that consumed at dinner; individuals with higher consumption of ultra-processed foods tended to consume even less vegetables at dinner.

**CONCLUSIONS:**

The consumption of vegetables in Brazil is insufficient, and this is worse among individuals with higher consumption of ultra-processed foods. The most frequent habit was to consume raw vegetables at lunch and with limited variety.

## RESUMO

**OBJETIVO:**

Caracterizar a aquisição domiciliar e o consumo alimentar individual de hortaliças no Brasil e analisar sua relação com o consumo de alimentos ultraprocessados.

**MÉTODOS:**

Foram utilizados dados de aquisição de alimentos para consumo no domicílio e de consumo alimentar individual da Pesquisa de Orçamentos Familiares 2008–2009. A Pesquisa de Orçamentos Familiares estudou a aquisição de alimentos de 55.970 domicílios e o consumo alimentar de 34.003 indivíduos com 10 anos ou mais de idade. Os alimentos de interesse neste estudo foram as hortaliças (excluindo raízes e tubérculos) e os alimentos ultraprocessados. A quantidade de hortaliças (gramas) adquiridas e consumidas foi descrita para o conjunto dos brasileiros e segundo quintos da participação calórica de alimentos ultraprocessados na alimentação. Para tanto, foram calculados os valores brutos e preditos, obtidos por modelos de regressão ajustados por variáveis sociodemográficas. Analisaram-se os tipos mais adquiridos de hortaliças (% na quantidade total) e, em relação ao consumo alimentar individual, a variedade de hortaliças consumidas (número absoluto), a participação (%) dos tipos de preparação culinária à base de hortaliças e os horários de consumo.

**RESULTADOS:**

A aquisição domiciliar média ajustada de hortaliças foi 42,9 g/*per capita*/dia. O consumo individual médio ajustado foi 46,1 g. Verificou-se relação inversa entre aquisição domiciliar e consumo individual de hortaliças e de alimentos ultraprocessados. Dez tipos de hortaliças respondem por mais de 80% da quantidade total habitualmente adquirida. A variedade consumida foi, em média, 1,08 tipo/*per capita*/dia. Cerca de 60% das hortaliças foram consumidas cruas, sendo a quantidade consumida no almoço duas vezes maior que aquela do jantar e indivíduos com maior consumo de alimentos ultraprocessados tenderam a consumir quantidade ainda menor de hortaliças no jantar.

**CONCLUSÕES:**

O consumo de hortaliças no Brasil é insuficiente, sendo pior entre indivíduos com maior consumo de alimentos ultraprocessados. O hábito mais frequente foi consumir hortaliças cruas, no almoço e com limitada variedade.

## INTRODUCTION

Fruits and vegetables are important sources of vitamins, minerals, fibers, and other bioactive compounds, in addition to presenting low energy density, which makes their consumption at adequate levels an important protective factor for morbidity (cardiovascular diseases, hypertension, diabetes, and some types of cancers) and mortality^1^ ^–^ ^3^. However, despite the international and national recommendations on the consumption and the variety of types and culinary preparations using these types of food^1^ ^,^ ^4^ ^,^ ^5^ ^,^ ^a^, their consumption remains below what is desired in most countries, and the scenario is even worse for vegetables when compared to fruits^6^ ^–^ ^8^.

In Brazil, between 1987–1988 and 2008–2009, there were some stability and low levels of purchase of fruits and vegetables, and there was even a small reduction in the consumption of vegetables. In 2008, the combined share of these types of food in the diet represented 2.9% of the total calories, and of this value 2.2% was for fruits and 0.7% for vegetables. However, in the same period, there was a marked decline in the participation of fresh food in the diet of the Brazilian population, together with a significant increase in the consumption of ultraprocessed foods, leading to a reduced overall quality of the diet in the country^9^.

Regarding the consumption of vegetables, only 35% of the adolescents and 38% of the adults (residents of the state capitals and the Federal District) reported regular consumption (five or more days of the week), while 20% and 8% reported not consuming them, respectively^b^ ^,^ ^c^. For comparison purposes, among Brazilian adolescents, the frequency of regular consumption of vegetables in 2012 was already lower than that found for confectionary (41.3%) and very similar to those observed for cookies (32.5%), crackers (35.1%), and soft drinks (33.2%)^10^.

Given this scenario, the Dietary Guidelines for the Brazilian Population recommends replacing the consumption of ultra-processed foods with natural or minimally processed foods. This strategy aims to improve effectively the quality of the diet^11^ ^,^ ^12^, valuing the variety of plant food available, respecting the biological, social, cultural, and food diversity of the country and exploring the possibilities of food combinations and culinary preparations^1^.

Given the context of the consumption of vegetables and ultra-processed foods in the country, the recommendations of the Dietary Guidelines for the Brazilian Population, and the scarcity of studies that explore the consumption of vegetables besides the adequacy in the amount and the regularity, this study aimed to characterize the consumption of vegetables in Brazil and analyze their relation with the consumption of ultra-processed foods.

## METHODS

### Data Source, Sample, and Data Collection

We used data from the Household Budget Survey (POF) carried out by the Brazilian Institute of Geography and Statistics (IBGE) between May 2008 and May 2009. It is a research with data representative of the Brazilian population, distributed in the five macro-regions and in the urban and rural areas of the country.

The 2008–2009 POF uses a complex sampling plan, by clusters, involving the geographic and socioeconomic stratification of all census tracts in the country, followed by random drawing of the tracts, in the first stage, and of the households, in the second stage. In total, 68,373 households were selected, of which 55,970 were effectively interviewed. The data was collected over a period of 12 months, in a uniform way in the strata, ensuring representativeness in the four quarters of the year^d^.

In this study, we used data related to the household purchase of food, registered by all the households sampled, and the individual food consumption, collected in a random subsample of the population (13,569 households, equivalent to 24.3% of the total, involving all individuals aged 10 years and over, amounting to 34,003 individuals).

We also used data on the household income *per capita* (R$/*per capita*/month), area (urban or rural), region of the household (North, Northeast, Midwest, Southeast, and South), date of birth (used to calculate the age and estimated in full years), and sex of the individuals studied.

### Purchase of Food for Consumption in the Household

We obtained the data on the purchase of food for the household consumption using the record of food and beverages purchased, written in a specific booklet by the residents of the households (or interviewer, when necessary) for seven consecutive days^d^. Given the short reference period used to record the food expenses in each household, we decided to use the household cluster as study units, defined based on the 550 sample strata, whose standard of annual food purchase may be known with greater accuracy. The average number of households studied in each stratum was 101.8, ranging from eight to 796 households.

The weekly amount of each food item, after excluding its inedible fraction, was divided by seven (number of days in the week) in order to express daily consumption values. Then, we converted the daily net amount purchased, in grams or milliliters, into energy for each food, using the Brazilian Food Composition Table (TACO)^e^ or, when the food was not present in that table, the food composition table of the United States Department of Agriculture (USDA), version 23^f^.

### Individual Food Consumption

Food consumption data were obtained from two daily diet records on non-consecutive days. Individuals aged 10 years and over living in the sampled households recorded all food and beverages consumed in a 24-hour period, indicating the amounts consumed in home measures and the form of preparation^g^. For this study, we considered only the consumption data of individuals who completed the two days of food record (n = 32,900).

We transformed the amount for each food or beverage, reported by the individual in home measurements, into grams or milliliters using the Table of Measures for Food Consumed in Brazil^h^. Then, we converted the amounts of food or beverages into energy based on the Table of Nutritional Composition of Food Consumed in Brazil^i^.

### Definition of the Study Indicators

The foods of interest in this study were vegetables (in all forms of preparation, excluding roots and tubers) and ultra-processed foods.

Regarding the vegetables, in relation to the purchase for household consumption, we considered all the vegetables purchased. In the case of individual food consumption data, in addition to the inclusion of the vegetables consumed (i) raw (e.g., in salads) and (ii) cooked, we decided to include culinary preparations based on vegetables, divided into: (iii) sautéed, garlic and oil or butter, (iv) with sauce or stew, (v) fried or breaded, (vi) grilled, baked, or barbecue, and (vii) soups. In these five culinary preparations, given the inclusion of other ingredients that affect the final weight of the preparation, we standardized the participation of the vegetables for each type of preparation to estimate the amount of vegetables consumed. In the case of soups, given the amount of liquid and other ingredients, we considered that the vegetables accounted for 25% of the total weight of the preparation^j^. For the other preparations, we used the percentage adopted in the POF^i^.

The ultra-processed food group was defined based on the NOVA classification^13^, namely: ultra-processed foods are ready-to-eat industrial formulations made entirely or predominantly of substances extracted from food (oils, fats, sugar, starch, proteins), derived from food constituents (hydrogenated fats, modified starch), or synthesized in the laboratory based on organic materials (dyes, flavorings, flavor enhancers, and various types of additives used to provide products with attractive sensory properties). This group includes the following food subgroups: crackers and chips; cakes, pies, and cookies; cereals; processed meats, hamburgers, and sausages; confectionaries (candies, button-shaped chocolates, chocolates, gelatin, puddings, and ice creams); fast food (hamburgers and cheeseburgers, hot dogs, fried and roasted savory snacks, and similar products); margarine; industrial sauces; sliced bread, hamburger buns, hot dog buns, and similar products; ready or semi-ready dishes (pizzas, frozen pasta or meat dishes, instant noodles, and powdered soups); ultra-processed cheeses; soft drinks and other sugary drinks.

### Data Analysis

Initially, the amounts of vegetables (gram/*per capita*/day) and ultra-processed foods (kcal/*per capita*/day) purchased for household and individual consumption were described for Brazil and according to sociodemographic characteristics.

We described the amounts (gram/*per capita*/day) of vegetables – purchased by the households and consumed by the individuals – for the entire Brazilian population and according to the quintiles of caloric intake of ultra-processed food in the diet. Then, we calculated the predicted (adjusted) values for household purchase and individual consumption of vegetables using multiple linear regression models, taking into account the sociodemographic characteristics. For the purchase of vegetables for household consumption, we considered the income *per capita* (in natural logarithm), area, and region; for the individual consumption of vegetables, we considered the income *per capita* (in natural logarithm), area, region, sex, and age.

We adopted an analogous adjustment procedure to describe the participation of the ten most purchased vegetables for household consumption in the total vegetables purchased in Brazil and in the groups with the lowest (1st quintile) and highest (5th quintile) intake of ultra-processed foods. We tested the difference in the participation of each type of vegetable between the ultra-processed food consumption groups (1st and 5th quintiles) using multiple linear regression models.

In relation to the individual food consumption of vegetables, we analyzed the variety of vegetables consumed (expressed in absolute numbers), the participation (%) of the types of culinary preparations based on vegetables in the total consumption of vegetables, and time of consumption. The adjusted variety of vegetables consumed per individual and the adjusted participation of the types of culinary preparations in the consumption were described for all Brazilians studied and for the Brazilians with the lowest (1st quintile) and highest (5th quintile) intake of ultra-processed foods. Furthermore, we also described the adjusted amount of vegetables consumed (in grams), according to the time of consumption, for all Brazilians studied and for the Brazilians with the lowest and highest intake of ultra-processed foods. We tested the difference between the ultra-processed food consumption groups (1st and 5th quintiles) for variety, each type of preparation, and each time of consumption using multiple linear regression models.

We performed all analyzes using the Stata software (StataCorp LP, College Station, Texas, United States), version 14.2, taking into account the complex design of the sample.

## RESULTS

The average amount of vegetables purchased for consumption in Brazilian households was 43.7 grams/*per capita*/day, and the average daily individual consumption was 49.2 grams/*per capita*/day. In relation to ultra-processed foods, average household purchase accounted for 18.0% of the total calories and individual consumption accounted for 20.5% (Table 1).

**Table 1:** Amount of vegetables (in grams) and ultra-processed foods (in kcal) purchased and consumed in Brazil, according to sociodemographic characteristics. Brazil, 2008–2009.

Variable		Household food purchase (n = 55,970 households)		Individual food consumption (n = 32,900 individuals)	
		Vegetables (g/ *per capita* /day) (95%CI)	Ultra-processed foods (kcal/ *per capita* /day) (95%CI)	Vegetables (g/ *per capita* /day) (95%CI)	Ultra-processed foods (kcal/ *per capita* /day) (95%CI)
Household income *per capita* (R$)					
	1st tercile	33.0 (31.0–34.9)	12.4 (11.7–13.2)	31.5 (30.2–32.8)	15.1 (14.6–15.5)
	2nd tercile	42.1 (37.8–46.2)	17.6 (16.5–18.8)	48.3 (46.7–50.0)	20.2 (19.7–20.8)
	3rd tercile	56.1 (52.0–60.1)	23.9 (22.8–24.9)	67.6 (64.9–70.3)	26.3 (25.7–26.9)
Area					
	Rural	39.2 (35.3–43.0)	10.6 (9.7–11.4)	41.3 (39.5–43.2)	12.7 (12.3–13.2)
	Urban	44.5 (41.8–47.2)	19.3 (18.5–20.2)	50.7 (49.4–52.1)	22.1 (21.7–22.5)
Region					
	North	31.9 (28.6–35.3)	11.7 (10.4–13.0)	28.8 (27.0–30.5)	14.8 (14.3–15.4)
	Northeast	37.2 (34.4–40.0)	14.6 (13.7–15.4)	30.5 (29.2–31.8)	14.9 (14.5–15.3)
	Southeast	45.6 (40.9–50.2)	20.2 (18.9–21.5)	56.4 (54.4–58.5)	23.6 (23.0–24.2)
	South	53.4 (49.4–57.4)	22.0 (20.6–23.3)	60.0 (60.1–68.0)	25.7 (25.0–26.4)
	Midwest	45.9 (40.8–51.0)	14.5 (13.2–15.8)	67.5 (64.1–70.8)	19.4 (18.4–20.3)
Sex					
	Male	-	-	47.6 (45.9–49.2)	19.2 (18.7–19.7)
	Female	-	-	50.6 (49.1–52.3)	21.8 (21.3–22.2)
Age (years)					
	10–19	-	-	31.6 (29.9–33.3)	26.8 (26.1–27.6)
	20–39	-	-	47.8 (45.8–49.7)	21.3 (20.8–21.9)
	40–59	-	-	57.6 (55.3–59.9)	17.2 (16.6–17.8)
	60 or more	-	-	63.8 (60.1–67.4)	15.0 (14.2–15.8)
Brazil		43.7 (41.4–46.0)	18.0 (17.1–17.8)	49.2 (48.0–59.3)	20.5 (20.2–20.8)

The amount of vegetables and ultra-processed foods related to household acquisition increases with the increase in income and differs for the regions: for vegetables, it was higher in the South, Southeast, and Midwest regions, and for ultra-processed foods, it was higher in the South and Southeast regions. For ultra-processed foods, we also observe that the amount purchased is higher in the urban area. In relation to the individual food consumption of vegetables and ultra-processed foods, we observed similar results for income, areas, and regions, and the only difference in relation to the acquisition was the greater consumption of vegetables in the urban area. In addition, women consume more ultra-processed foods, and age was inversely related to the consumption of ultra-processed foods and directly related to the consumption of vegetables (Table 1).

After adjusting for the sociodemographic variables, the average amount of vegetables purchased for consumption in Brazilian households was 42.9 g/*per capita*/day. In the adjusted analyses, we observed an inverse relation between the purchase of vegetables and ultra-processed foods, that is, households with higher caloric intake of ultraprocessed foods purchased fewer vegetables. After adjusting for sociodemographic variables, we observed an average value of 46.1 g/*per capita*/day for individual food consumption. There was also an inverse relation between the consumption of vegetables and ultra-processed foods (Table 2).

**Table 2:** Amount (in grams) of vegetables purchased and consumed in Brazil and according to quintiles of caloric intake of ultra-processed foods: crude and adjusted values. Brazil, 2008–2009.

Amount of vegetables (g/ *per capita* /day)		Brazil	Quintiles of caloric intake of ultra-processed foods (% of the total of energy)				
Household purchase			1st (9.0)	2nd (14.4)	3rd (17.4)	4th (21.5)	5th (27.4)
	Crude values	43.7	37.1	38.7	43.7	43.3	56.0^c^
	Adjusted values^a^	42.9	46.9	44.4	41.9	39.4	36.9^c^
Individual consumption			1st (1.8)	2nd (9.6)	3rd (17.9)	4th (28.8)	5th (49.2)
	Crude values	49.2	42.5	53.2	55.8	53.2	41.2
	Adjusted values^b^	46.1	49.4	47.7	45.9	44.1	42.3^c^

a Model adjusted for income *per capita* (in log), area, and region.

b Model adjusted for income *per capita* (in log), area, region, sex, and age.

c p < 0.05 for linear trend, obtained by multiple linear regression model.

Ten types of vegetables accounted for 83% of the amount (in grams) usually purchased by Brazilians, and this variety was not affected by the participation of ultra-processed foods in the diet (82.7% in the case of the first quintile of caloric intake of ultra-processed foods *versus* 84.1% in the fifth quintile). The vegetables with the highest participation in the household purchase were: tomato (29.2% of the total vegetables purchased), onion (19.4%), carrot (8.1%), cabbage (5.4%), lettuce (4.8%), pumpkin (4.0%), chayote (3.4%), bell pepper (3.2%), garlic (3.1%), and beet (2.4%). Families with lower consumption of ultra-processed foods consumed more cabbage, pumpkin, and beet and consumed less onion, carrot, chayote, and bell pepper in the total vegetables purchased (Table 3).

**Table 3:** Participation of vegetables in the household purchase in Brazil and by Brazilians with the lowest and highest participation of ultra-processed foods in the diet. Brazil, 2008–2009.

Vegetables	Participation (%) in the total amount (grams) of vegetables purchased for consumption in households^a^		
	Brazil	1st quintile of caloric intake of ultra-processed foods	5th quintile of caloric intake of ultra-processed foods
Tomato	29.2	28.9	30.7
Onion	19.4	19.2	22.1^b^
Carrot	8.1	6.3	8.8^b^
Cabbage	5.4	6.5	3.9^b^
Lettuce	4.8	4.2	4.3
Pumpkin	4.0	5.9	2.6^b^
Chayote	3.4	3.0	3.8^b^
Bell pepper	3.2	2.7	3.5^b^
Garlic	3.1	3.3	2,6
Beet	2.4	2.8	1.9^b^
Total	82.9	82.7	84.1

a Values adjusted for income *per capita*, area, and region.

b p < 0.05 of the linear trend among the quintiles of ultra-processed food consumption, obtained by multiple linear regression model.

Regarding individual food consumption, 42.1% of the Brazilians studied did not report consumption of vegetables. Among those who reported having consumed vegetables, the adjusted variety of vegetables was 1.08 type/day for the entire population. Individuals with lower consumption of ultra-processed foods consumed 1.14 type/day of vegetables, and those with higher consumption of ultra-processed foods consumed 1.05 type/day (p = 0.008) (data not shown).

The most consumed types of vegetable culinary preparations were raw (59.2%), cooked (17.4%), soup (15.4%), and sautéed (6.8%). Among the individuals with the lowest and highest consumption of ultra-processed foods, we only observed a difference for soup, the most consumed preparation by the group with the lowest consumption of ultra-processed foods (Table 4).

**Table 4:** Types of preparations based on vegetables consumed by all Brazilians and among those with the lowest and highest consumption of ultra-processed foods. Brazil, 2008–2009.

Type of preparation	Participation (%) in the total amount of vegetables consumed^a^		
	Brazil	1st quintile of caloric intake of ultra-processed foods	5th quintile of caloric intake of ultra-processed foods
Raw	59.2	54.2	62.8
Cooked	17.4	19.4	16.6
Soup	15.4	18.6	12.6^b^
Sautéed, garlic and oil or butter	6.8	7.0	6.3
With sauce or stew	0.6	0.3	0.9
Fried or breaded	0.6	0.5	0.7
Grilled, roasted, or barbecue	0.0	0.0	0.1

a Values adjusted for income *per capita* (in log), area, region, sex, and age.

b p < 0.05 of the linear trend among the quintiles of ultra-processed food consumption, obtained by multiple linear regression model.

Finally, we verified that the consumption of vegetables is concentrated in two periods of the day, equivalent to lunch and dinner. However, the amount consumed between 11 am and 2 pm is double the amount consumed between 7 pm and 10 pm. Individuals with lower consumption of ultra-processed foods consumed less vegetables between 1 pm and 2 pm (p < 0.05) and consumed more vegetables between 6 pm and 8 pm (p < 0.001), when compared to those with higher consumption of ultra-processed foods (Figure).

**Figure:**
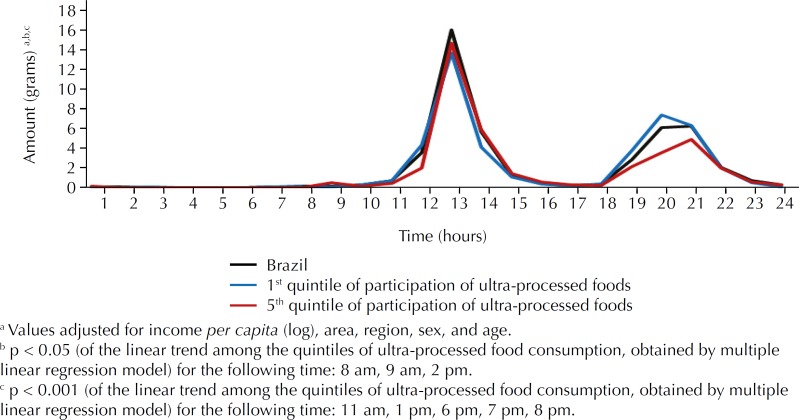
Amount of vegetables consumed (in grams), according to time of consumption, for all Brazilians and among those with the lowest (1st quintile of caloric intake) and highest (5th quintile of caloric intake) consumption of ultra-processed foods. Brazil, 2008–2009.

## DISCUSSION

The analysis of the data on food purchase for household and individual consumption of the Brazilian population, based on data of the 2008–2009 POF, showed insufficient consumption of vegetables and their inverse relation with the consumption of ultra-processed foods. We can observe that ten types of vegetables account for more than 80% of the total amount usually purchased. In individual consumption, there is a relative monotony, since the predicted variety of vegetables was 1.08 type/day. Approximately 60% of the vegetables are eaten raw, and the amount consumed at lunch is twice as large as that consumed at dinner. The amount of vegetables consumed at lunch was similar among the different levels of consumption of ultra-processed foods; at dinner, individuals with lower consumption of ultra-processed foods tended to consume more vegetables.

The inverse relation between the consumption of vegetables and ultra-processed foods reinforces the already extensive list of adverse effects related to the consumption of these products. From this, we infer that, in addition to the harms directly linked to the consumption of ultra-processed foods (from their negative nutritional attributes), this consumption also indirectly harms the diet given their ability to displace and interfere with the consumption of healthy food. This result was expected in view of the body of evidence available regarding the risks of consuming ultraprocessed foods^15^ ^–^ ^18^. Studies evaluating the impact of ultra-processed foods on the nutritional profile of the diet of Brazilians have identified a worsening in the quality of the diet (higher energy density; more free sugar and saturated and trans fats; lower fiber density, protein content, and micronutrient intake) with increased consumption of ultra-processed foods in it^11^ ^,^ ^12^.

Despite the wide variety of vegetables found in Brazil^a^, we observed a limited variation in this study, as ten types of vegetables accounted for 83% of the amount usually consumed. In relation to the number of vegetables consumed per day, it was slightly higher than one in all analyzed situations, which reinforces the low variety in the consumption. Despite the importance of varied consumption for the quality of the diet, this subject has been little explored in the literature^14^ ^,^ ^k^, and we found no Brazilian study describing the consumption of different types of vegetables or even interventions focused on the increase of variety in the consumption of vegetables.

Regarding the culinary preparation, despite the versatility of the vegetables^1^ ^,^ ^a^ more than half was consumed raw in our study. Furthermore, studies that describe the different types of preparations consumed are scarce, which represents an important gap in the literature. A national survey conducted with adolescents has found that 27% of these young persons had a regular consumption of raw vegetables and 14% consumed them cooked; however, other types of preparations were not evaluated^c^. On the other hand, the preparation of vegetables has been approached in studies that evaluate the impact of different preparation techniques on nutritional composition^19^.

The highest consumption of vegetables occurred during lunch (between 12 am and 2 pm), which differs from the North American and Norwegian populations, who consume these types of food mainly at dinner^6^ ^,^ ^20^. This result reflects the high prevalence of the consumption of complete meals at lunch in Brazil, and dinner is the meal usually replaced by snacks. The data of the VIGITEL, obtained from a sample of adult individuals (≥ 18 years old) living in the Brazilian state capitals and the Federal District in 2014, indicate that 16.5% of the study population reported replacing dinner with snacks (meals with high participation of ultraprocessed foods) and only 1.4% reported doing so during lunch^l^. The higher replacement of traditional meals for snacks at dinner explains, albeit in part, the reason why individuals who consume more ultra-processed foods have a lower consumption of vegetables in the evening.

Considering the findings of our study, individual, collective, and structure strategies are needed to improve the quality of the diet of Brazilians. As an individual strategy, we highlight the need to include vegetables at dinner, replacing ultra-processed foods^20^. In addition, interventions should focus on the diversification of vegetables and the forms of culinary preparation used. These strategies are closely related to the recommendations of the Dietary Guidelines, which points out the importance of the variety in the diet and the development of culinary skills in the promotion of healthy eating^1^.

From the collective point of view, considering the increasingly frequent practice of eating outside the home, scenarios of collective meals, especially in the organizational environment, can be considered strategic to ensure the supply of vegetables and to favor their consumption, as it has been observed in some workplaces, universities, and schools^21^ ^,^ ^m^ ^,^ ^n^.

Furthermore, structure strategies related to the production and distribution of vegetables, such as the Brazilian Food Acquisition Program^o^, are relevant, as there are significant disparities in the access to these types of food^22^ ^–^ ^24^. In addition, our study did not aim to identify factors associated with vegetable consumption in Brazil; however, issues related to the lack of physical or financial access to these types of food are often listed as determinants for their low consumption^25^ ^,^ ^26^.

We mention the option of not analyzing fruits and vegetables together. We made this decision because these types of food, although treated together in nutritional recommendations, tend to differ in relation to the amount consumed^9^ and the barriers mentioned to increase consumption^27^. Considering that interventions focused on the greater consumption of fruits and vegetables need different outlooks, we can justify the need for more detailed analyses for each of these groups.

Dietary records, such as those used in this study, have potential biases, namely: underestimation of the food consumed, change in the habitual consumption on the days of the registration, and differences between the recipes of the preparations practiced by the individuals and the standardized recipes used. In order to minimize these biases, the data collection instrument used was pre-tested and validated, and quality control procedures were used during data collection; in addition, inconsistent records were excluded and replaced with imputed values^h^. Regarding the quantification of vegetables, potential errors were minimized with the use of standardization for their participation in the different types of culinary preparations. In addition, since the instrument was not designed to evaluate food consumption according to the extent and purpose of its industrial processing, some items may have been misclassified. These errors are most likely to be found in items such as pizzas, sweets, and fruit juices, which can be classified as either preparations or ultra-processed foods. In the absence of information in the records allowing a distinction between culinary preparations and ultra-processed foods (given on the recipe or the brand of the product), we chose to classify them in their most frequent form, considering the cases in which there was information.

On the other hand, because of the use of data on household purchase and individual food consumption representative of the Brazilian population, this study contributes in an important way to give a panorama, with different perspectives, on the national practices related to the consumption of vegetables and their relation with the consumption of ultra-processed foods.

The differences in the amount observed for vegetables purchased and consumed may refer to the preparation technique used, the incorporation of oils and fats, some possible waste of vegetables, or the consumption of food outside the home, measured only for individual consumption. In addition, data on food purchase allow the identification of vegetables commonly used to season food, such as onions and garlic, which hardly appear alone in individual food consumption data. Although the literature points out similarities between acquisition and consumption data^28^ ^–^ ^30^, we decided to present the two estimates, as they offer information regarding different dimensions of the consumption of vegetables. While the data on household acquisition shows the consumption intention and the usual variety (free of seasonal influence), the data on individual consumption provides information on the amount actually consumed, the preparations consumed, and the time of consumption.

We conclude that the consumption of vegetables in Brazil is insufficient, and this is worse among individuals with higher consumption of ultra-processed foods. The most frequent habit was to consume raw vegetables at lunch and with limited variety.

## Footnotes

a Ministério da Saúde (BR), Secretaria de Atenção à Saúde, Departamento de Atenção Básica. Alimentos regionais brasileiros. 2.ed. Brasília (DF); 2015 [cited 2017 Mar 2]. Available from: http://bvsms.saude.gov.br/bvs/publicacoes/alimentos_regionais_brasileiros

b Ministério da Saúde (BR), Secretaria de Vigilância em Saúde, Departamento de Vigilância de Doenças e Agravos não Transmissíveis e Promoção da Saúde. Vigitel Brasil 2014: vigilância de fatores de risco e proteção para doenças crônicas por inquérito telefônico. Brasília (DF); 2015 [cited 2017 Mar 2]. Available from: http://bvsms.saude.gov.br/bvs/publicacoes/vigitel_brasil_2014.pdf

c Instituto Brasileiro de Geografia e Estatística. Pesquisa Nacional de Saúde do Escolar 2012. Rio de Janeiro: IBGE; 2013.

d Instituto Brasileiro de Geografia e Estatística. Pesquisa de Orçamentos Familiares 2008-2009: análise da disponibilidade domiciliar de alimentos e do estado nutricional no Brasil. Rio de Janeiro: IBGE; 2010.

e Universidade Estadual de Campinas, Núcleo de Estudos e Pesquisas em Alimentação. Tabela Brasileira de Composição de Alimentos – TACO. Campinas: UNICAMP; 2004.

f United States, Department of Agriculture. USDA National Nutrient Database for Standard Reference. Release 23. Beltsville (MD): Nutrient Data Laboratory; 2010.

g Instituto Brasileiro de Geografia e Estatística. Análise do consumo alimentar pessoal no Brasil. Instituto Brasileiro de Geografia e Estatística. Rio de Janeiro; 2011.

h Instituto Brasileiro de Geografia e Estatística. Tabela de medidas referidas para os alimentos consumidos no Brasil. Rio de Janeiro: IBGE; 2011.

i Instituto Brasileiro de Geografia e Estatística. Tabelas de composição nutricional dos alimentos consumidos no Brasil. Rio de Janeiro: IBGE; 2011.

j Bombem KCM, Canella DS, Bandoni DH, Jaime PC. Manual de medidas caseiras e receitas para cálculos dietéticos. São Paulo: M. Books do Brasil; 2012.

k United States, Department of Agriculture, Economic Research Service. Selected charts from Ag and Food Statistics Charting the Essentials, 2017. Washington, DC: USDA; 2017.

l Ministério da Saúde (BR), Secretaria de Vigilância em Saúde. Vigitel Brasil 2014: vigilância de fatores de risco e proteção para doenças crônicas por inquérito telefônico. Brasília (DF); 2015 [cited 2017 Mar 2]. Available from: http://svs.aids.gov.br/bases_vigitel_viva/

m Perez PMP. Impacto da implementação do Restaurante Universitário na alimentação de estudantes da Universidade do Estado do Rio de Janeiro [these]. Rio de Janeiro: Universidade do Estado do Rio de Janeiro; 2015.

n Locatelli NT. Consumo da alimentação oferecida nas escolas públicas brasileiras e sua relação com fatores sociodemográficos e práticas alimentares: resultados da Pesquisa Nacional de Saúde do Escolar 2012 [dissertation]. Santos: Instituto de Saúde e Sociedade da Universidade Federal de São Paulo; 2016.

o Casa Civil (BR), Secretaria Especial de Agricultura Familiar e do Desenvolvimento Agrário. Programa de Aquisição de Alimentos. Brasília (DF); 2003 [cited 2017 Jan 31]. Available from: http://www.mda.gov.br/sitemda/secretaria/saf-paa/sobre-o-programa

## References

[1] Ministério da Saúde (BR), Secretaria de Atenção à Saúde, Departamento de Atenção Básica (2014). Guia alimentar para a população brasileira.

[2] Wang X, Ouyang Y, Liu J, Zhu M, Zhao G, Bao W (2014). Fruit and vegetable consumption and mortality from all causes, cardiovascular disease, and cancer: systematic review and dose-response meta-analysis of prospective cohort studies. BMJ.

[3] Rezende LFM, Azeredo CM, Canella DS, Luiz OC, Levy RB, Eluf J (2016). Coronary heart disease mortality, cardiovascular disease mortality and all-cause mortality attributable to dietary intake over 20 years in Brazil. Int J Cardiol.

[4] World Health Organization (2002). Diet, nutrition and the prevention of chronic diseases.

[5] Australian Government, National Health and Medical Research Council (2013). Australian dietary guidelines.

[6] Satia J, Kristal A, Patterson R, Neuhouser ML, Trudeau E (2002). Psychosocial factors and dietary habits associated with vegetable intake. Nutrition.

[7] Abe SK, Stickley A, Roberts B, Richardson E, Abbott P, Rotman D (2013). Changing patterns of fruit and vegetable intake in countries of the former Soviet Union. Public Health Nutr.

[8] Micha R, Khatibzadeh S, Shi P, Andrews KG, Engell RE, Mozaffarian D (2015). Global, regional and national consumption of major food groups in 1990 and 2010: a systematic analysis including 266 country-specific nutrition surveys worldwide. BMJ Open.

[9] Martins APB, Levy RB, Claro RM, Moubarac J, Monteiro CA (2013). Participação crescente de produtos ultraprocessados na dieta brasileira (1987-2009). Rev Saude Publica.

[10] Azeredo CM, Rezende LFM, Canella DS, Claro RM, Castro IRR, Luiz OC (2015). Dietary intake of Brazilian adolescents. Public Health Nutr.

[11] Louzada MLC, Martins APB, Canella DS, Baraldi LG, Levy RB, Claro RM (2015). Alimentos ultraprocessados e perfil nutricional da dieta no Brasil (2008-2009). Rev Saude Publica.

[12] Louzada MLC, Martins APB, Canella DS, Baraldi LG, Levy RB, Claro RM (2015). Impacto de alimentos ultraprocessados sobre o teor de micronutrientes da dieta no Brasil. Rev Saude Publica.

[13] Monteiro CA, Cannon G, Moubarac JC, Levy RB, Louzada MLC, Jaime PC (2018). The UN Decade of Nutrition, the NOVA food classification and the trouble with ultra-processing. Public Health Nutr.

[14] Oude Griep LM, Verschuren WM, Kromhout D, Ocké MC, Geleijnse JM (2012). Variety in fruit and vegetable consumption and 10-year incidence of CHD and stroke. Public Health Nutr.

[15] Canella DS, Levy RB, Martins APB, Claro RM, Moubarac JC, Baraldi LG (2014). Ultra-processed food products and obesity in Brazilian households (2008-2009). PLoS One.

[16] Rauber F, Campagnolo PD, Hoffman DJ, Vitolo MR (2015). Consumption of ultra-processed food products and its effects on children's lipid profiles: a longitudinal study. Nutr Metab Cardiovasc Dis.

[17] Mendonça RD, Lopes ACS, Pimenta AM, Gea A, Martinez-Gonzalez MA, Bes-Rastrollo M (2017). Ultra-processed food consumption and the incidence of hypertension in a Mediterranean cohort: The Seguimiento Universidad de Navarra Project. Am J Hypertens.

[18] Mendonça RD, Pimenta AM, Gea A, Fuente-Arrillaga C, Martinez-Gonzalez MA, Lopes ACS (2016). Ultra-processed foods consumption and risk of overweight and obesity: The University of Navarra Follow-Up (SUN) cohort study. Am J Clin Nutr.

[19] Murador DC, Cunha DT, Rosso VV (2014). Effects of cooking techniques on vegetable pigments: a metaanalytic approach to carotenoid and anthocyanin levels. Food Res Int.

[20] Myhre JB, Løken EB, Wandel M, Andersen LF (2015). Meal types as sources for intakes of fruits, vegetables, fish and whole grains among Norwegian adults. Public Health Nutr.

[21] Bandoni DH, Sarno F, Jaime PC (2011). Impact of an intervention on the availability and consumption of fruits and vegetables in the workplace. Public Health Nutr.

[22] Jaime PC, Machado FMS, Westphal MF, Monteiro CA (2006). Impact of a community-based intervention to increase fruit and vegetable intake among lowincome families from São Paulo, Brasil. Rev Chil Nutr.

[23] Duran AC, Diez-Roux AV, Latorre MRDO, Jaime PC (2013). Neighborhood socioeconomic characteristics and differences in the availability of healthy food stores and restaurants in Sao Paulo, Brazil. Health Place.

[24] Pessoa MC, Mendes LL, Gomes CS, Martins PA, Velasquez-Melendez G (2015). Food environment and fruit and vegetable intake in a urban population: a multilevel analysis. BMC Public Health.

[25] Claro RM, Monteiro CA (2010). Renda familiar, preço de alimentos e aquisição domiciliar de frutas e hortaliças no Brasil. Rev Saude Publica.

[26] Chor D, Cardoso LO, Nobre AA, Griep RH, Fonseca MJM, Giatti L (2016). Association between perceived neighbourhood characteristics, physical activity and diet quality: results of the Brazilian Longitudinal Study of Adult Health (ELSA-Brasil). BMC Public Health.

[27] Glasson C, Chapman K, James E (2011). Fruit and vegetables should be targeted separately in health promotion programmes: differences in consumption levels, barriers, knowledge and stages of readiness for change. Public Health Nutr.

[28] Becker W (2001). Comparability of household and individual food consumption data: evidence from Sweden. Public Health Nutr.

[29] Naska A, Vasdekis VGS, Trichopoulou A (2001). A preliminary assessment of the use of household budget survey data for the prediction of individual food consumption. Public Health Nutr.

[30] Claro RM, Jaime PC, Lock K, Fisberg RM, Monteiro CA (2010). Discrepancies among ecological, household, and individual data on fruits and vegetables consumption in Brazil. Cad Saude Publica.

